# Oxygen vacancies engineering in electrocatalysts nitrogen reduction reaction

**DOI:** 10.3389/fchem.2022.1039738

**Published:** 2022-10-12

**Authors:** Haijiang Zhu, Chao Wang, Yuling He, Yi Pu, Peiwen Li, Liang He, Xianglan Huang, Wu Tang, Hui Tang

**Affiliations:** ^1^ School of Materials and Energy, University of Electronic Science and Technology of China, Chengdu, China; ^2^ Institute of Machinery Manufacturing Technology, China Academy of Engineering Physics, Mianyang, China; ^3^ School of Mechanical Engineering, Sichuan University, Chengdu, China; ^4^ School of New Energy Materials and Chemistry, Leshan Normal University, Leshan, China

**Keywords:** electrocatalysts, nitrogen reduction reaction, oxygen vacancies engineering, density functional theory, synthesis methods

## Abstract

Ammonia is important, both as a fertilizer and as a carrier of clean energy, mainly produced by the Haber-Bosch process, which consumes hydrogen and emits large amounts of carbon dioxide. The ENRR (Electronchemical Nitrogen Reduction Reaction) is considered a promising method for nitrogen fixation owing to their low energy consumption, green and mild. However, the ammonia yield and Faraday efficiency of the ENRR catalysts are low due to the competitive reaction between HER and NRR, the weak adsorption of N2 andthe strong N≡N triple bond. Oxygen vacancy engineering is the most important method to improve NRR performance, not only for fast electron transport but also for effective breaking of the N≡N bond by capturing metastable electrons in the antibonding orbitals of nitrogen molecules. In this review, the recent progress of OVs (oxygen vacancies) in ENRR has been summarized. First, the mechanism of NRR is briefly introduced, and then the generation methods of OVs and their applicationin NRR are discussed, including vacuum annealing, hydrothermal method, hydrogen reduction, wet chemical reduction, plasma treatment and heterogeneous ion doping. Finally, the development and challenges of OVs in the field of electrochemical nitrogen fixation are presented. This review shows the important areas of development of catalysts to achieve industrially viable NRR.

## 1 Introduction

The continued rise of the global population and excessive use of fossil fuels has led to severe environmental issues and an energy crisis. As the main constituent of air, inert nitrogen gas can be converted into ammonia, which has a large variety of applications in the industry (Galloway et al., 2008; Zamfirescu and Dincer, 2008). Nearly 80% of the ammonia produced is utilized for fertilizers, making it a significant agricultural chemical. Additionally, it can be used as a potential carrier of green fuel (Galloway et al., 2004; Christensen et al., 2006; Kitano et al., 2012; Chen et al., 2018; Li et al., 2022b). Currently, the Haber-Bosch process is used for the production of ammonia from nitrogen and hydrogen, which was invented in the early 20th century (Tanabe and Nishibayashi, 2013; Liu, 2014). However, owing to the high bond energy, lack of dipole moment, and low polarization of the molecular structure of N_2_, leads to the production of Haber-Bosch process must be carried out at high pressures (150–300 ATM) and high temperatures (400–600°C), making it an energy-intensive process that accounts for about 1–2% of the world’s yearly energy supplies (Chirik, 2009; van der Ham et al., 2014; Singh et al., 2017; Sun et al., 2017; Guo et al., 2018; Chen et al., 2019; Song et al., 2019). There is an urgent need for researchers to find a viable and novel method of nitrogen fixation.

Recently, many methods have been proposed for nitrogen fixation in ambient circumstances, such as biochemical catalysis, photocatalysis, and electrocatalysis (Brown et al., 2016; Milton et al., 2016; Kyriakou et al., 2017; Cao and Zheng, 2018; Cui et al., 2018; Guo W et al., 2019). Among that the ENRR has been singled out as a promising method, because of their environment friendly, low pressure and moderate temperature (Zhang et al., 2019a; Zhang et al., 2019b; Li et al., 2019; Yu et al., 2019; Lazouski et al., 2020; Yang et al., 2020). However, the completion of NRR and hydrogen evolution reaction (HER) leads to the low Faraday efficiency and low ammonia yield, which limits its application (Hao et al., 2019; Qiu et al., 2019; Zhao et al., 2019). It is well-known that high Faraday efficiency and ammonia yield are requisite conditions for industrial applications of electrocatalytic reactions. Therefore, designing and producing environmentally friendly catalysts by an efficient process with low energy consumption and minimal pollution is crucial.

Oxygen vacancies engineering strategies as an effective method to improve NRR performance can tune the electronic structure and ensure successful reaction between intermediates, resulting in excellent chemical and physical properties as well as higher activity and selectivity (Yan et al., 2017; Xu et al., 2021; Gao et al., 2022; Ji et al., 2022). In recent studies, the design of catalyst materials with OVs for electrochemical NRR has drawn significant research attention (Zhang et al., 2018; Liu et al., 2019; Yan D. F et al., 2019; Zhang S et al., 2019; He et al., 2021). Moreover, the introduction of OVs in electrocatalysts has been extensively employed in NRR because a large number of stable metal oxide catalysts provide a variety of carriers for OVs enriched with different structures (Hirakawa et al., 2017; Xu et al., 2019; Li P. S et al., 2020; Liu et al., 2021b). For instance, Han et al. thoroughly investigated the ENRR performance and catalysis mechanism of titanium dioxide with various OVs concentrations by theoretical calculations and experiments, including strict control of the annealing temperature during the preparation process (Han et al., 2019). Accordingly, In-depth exploration of the effect of OVs on ENRR is essential to guide the design of catalysts with better catalytic performance.

In this review, we provide an overview of the most recent developments in utilizing OVs for developing catalysts for electrocatalytic nitrogen fixation. First, we briefly introduce the mechanism of electrocatalytic nitrogen fixation. We additionally summarize OVs generating methods and their applications for ENRR. Finally, the future development and possible challenges of OVs in the field of ENRR are discussed.

## 2 NRR mechanism

The complexity of the ENRR process and the catalyst’s shape, microstructure, electronic structure, and density of active sites influence the effectiveness of the catalyst. Moreover, inefficient reactions also occur since most electrons unite with protons to generate hydrogen, which is the largest competitive reaction in ENRR. Therefore, it is essential to have an in-depth understanding of the NRR process.

Adsorption and activation of N_2_ on the catalyst surface, along with the associated electron conversion and proton adsorption, are the first steps in the electrochemical reduction of N_2_ to NH_3_. This reaction is quite difficult for the following reasons: 1) the robust triple bond of the inert N_2_ molecule (Singh et al., 2017), 2) no permanent dipole, 3) a huge energy gap 10.82 eV between the highest occupied and lowest unoccupied molecular orbitals (Yan X et al., 2019), and 4) high ionization potential (15.58 eV) and low electron affinity (−1.9 eV) of the N_2_ molecule (Jia and Quadrelli, 2014; van der Ham et al., 2014). Consequently, A viable NRR catalyst requires modest binding to intermediate species and a high activation capacity relative to N_2_ (Wang et al., 2017).

**FIGURE 1:**
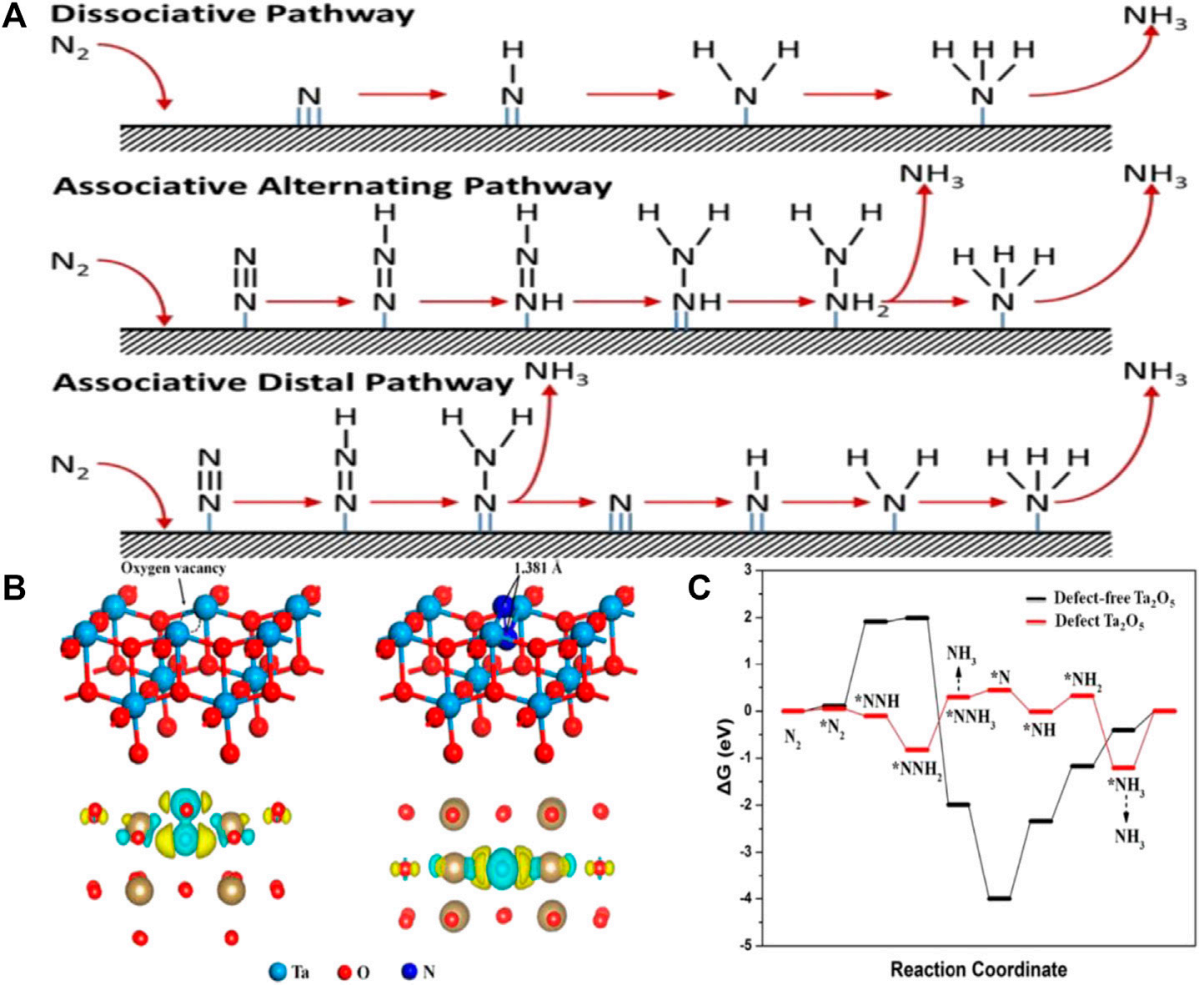
**(A)** Possible reaction mechanisms for the NRR to form NH_3_. (Shipman and Symes, 2017) with permission from Copyright 2016 Elsevier B.V. **(B)** Model of the (001) surface of Ta_2_O_5_ with an O-vacancy site, the adsorption geometry of N_2_ on the O-vacancy site of the Ta_2_O_5_ (001) surface, Side and top view of charge density difference of the N_2_ adsorbed (001) surface. **(C)** DFT-calculated NRR reaction pathways and the corresponding energy changes on defect Ta_2_O_5_ and defect-free Ta_2_O_5_. (Fu et al., 2019) with permission from Copyright 2019 American Chemical Society.

According to the intermediates involved and energy consumption, the NRR mechanism can be theoretically separated into dissociative and associative mechanisms (Figure 1A) (Shipman and Symes, 2017). The N≡N bond is first broken by the dissociative mechanisms before hydrogenation, and then individual N atoms are adsorbed onto the catalyst surface and hydrogenated to form NH_3_. The Haber-Bosch process follows the dissociative mechanism (Wan et al., 2019). The dissociative mechanism involves overcoming the high cleavage energy of the thermodynamic N≡N bond, making NRR unfavorable under ambient conditions.

In contrast to the dissociative pathway, the N≡N triple bond partially breaks in the associative mechanism, before the hydrogenation of N atoms takes place. The associative mechanism can be further classified into the distal path and the alternating path based on the sequence in which H atoms are added to the two distinct N atoms (Shipman and Symes, 2017). The distal N atom in the distal pathway first adsorbs the H atom, and subsequently hydrogenates until forming and releasing the ammonia molecule. Then another N atom is hydrogenated to release ammonia. The alternating path uses the alternating addition of H atoms to two different N atoms until one of them converts to NH_3_ and the N≡N bond is broken (Guo X et al., 2019).

In comparison to conventional catalysts, the introduction of OVs into a catalyst can increase the number of active sites for NRR by altering the electronic structure and surface properties. For example, Density functional theory (DFT) computations were carried out by Fu et al. on facets of Ta_2_O_5_ (001) with OVs (Fu et al., 2019). The localized electrons made the Ta ions reduce, leading to an increase in the Bader charge of the two connected Ta atoms, from 2.45 e to 2.92 and 3.00 e, as shown in Figure 1B. Meanwhile, the two partly reduced Ta atoms near the OVs swapped electrons and absorbed N_2_. Their accessible d orbitals were used by the N-N π antibonding orbital to acquire electrons, which contributed to activating and adsorbing the N_2_ molecule. The bond length of the successfully activated N_2_ molecule increased (to 1.381 Å) due to the transfer of electrons from the Ta atom to the adsorbed N_2_, which contrasts with 1.098 Å in free N_2_. Moreover, the OV-containing Ta_2_O_5_ adsorbed N_2_ more easily as shown in Figure 1C. Additionally, the hydrogenation of defect-free Ta_2_O_5_ had a large energy barrier. Therefore, OVs could enhance the catalyst’s NRR catalytic performance.

## 3 Methods to generate OVs and applications in ENRR

### 3.1 Thermal annealing in an oxygen-deficient environment

A widely used method to generate OVs is annealing oxygen-containing compounds at high temperatures under anoxic conditions (e.g., He, N_2_ and Ar) or vacuum. In the annealing process, the relative concentration of VOs can be adjusted by controlling the inert gas flow rate, final temperature, heating rate, annealing time and cooling rate (Sarkar and Khan, 2019; Wang L. et al., 2019; Zhu et al., 2019).

**FIGURE 2:**
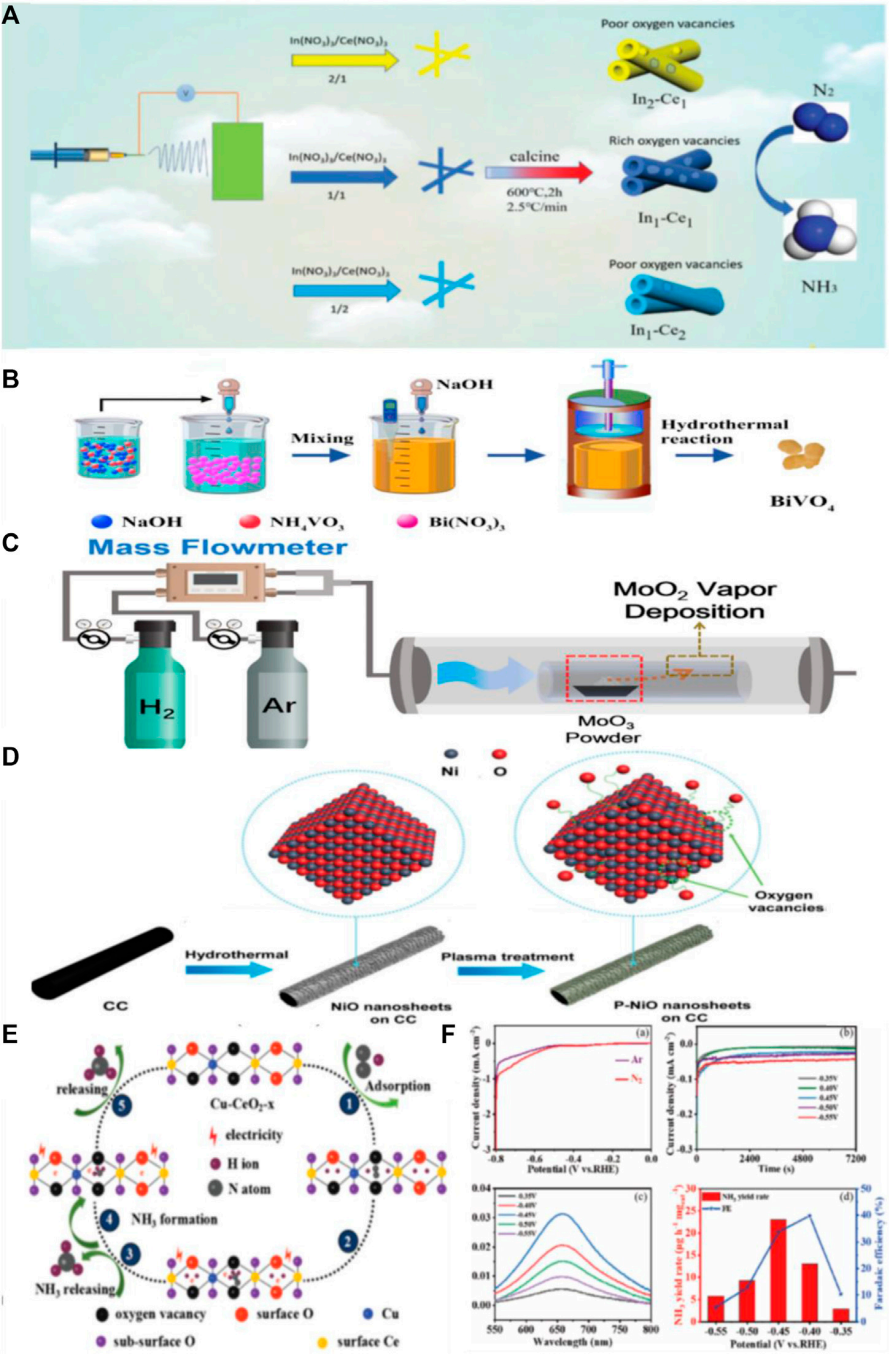
**(A)** The schematic diagram of the catalyst preparation and the illustration of the NRR on VO rich In_2_O_3-x_/CeO_2-y_. (Wang et al., 2020) with permission from 2020 Royal Society of Chemistry. **(B)** Schematic illustration of the preparation of BiVO_4_ by hydrothermal method. (Yao et al., 2019) with permission from 2018 WILEY-VCH Verlag GmbH and Co. KGaA, Weinheim. **(C)** Schematic of the preparation of MoO_2_ nanosheets. (Zhang G et al., 2019) with permission from Copyright 2019 Elsevier Ltd. **(D)** Schematic illustration of the synthesis process of P–NiO/CC. (Wang Y. et al., 2019) with permission from Copyright 2020 Royal Society of Chemistry. **(E)** The proposed NRR pathway for the NH_3_ synthesis on the Cu-CeO_2_-3.9 catalyst. (Zhang S et al., 2019) with permission from Copyright 2019 Royal Society of Chemistry. **(F)** NRR performance of P-KNO after electrolysis at different potentials. (Fan et al., 2022) with permission from Copyright 2022 Royal Society of Chemistry.

Wang et al. obtained In_2_O_3-x_/CeO_2-y_ nanotubes rich in OVs by electrostatic spinning followed by vacuum annealing (Figure 2A) (Wang et al., 2020). According to the XPS spectra, the sample In_1_-Ce_1_, with a raw material ratio of 1:1, showed the highest concentration of OVs. With the formation of OVs, the electrochemical properties were effectively optimized, and the kinetics of NRR and electron conduction capacity were significantly enhanced, leading to a Faraday efficiency of 16.1% and NH_3_ yield of 26.1 μg h^−1^ mg_cat_^−1^. Luo et al. synthesized MOF-derived N-doped carbon/Co_3_O_4_ nanocomposites (Co_3_O_4_@NCs) containing OVs by vacuum annealing (Luo et al., 2019). Co_3_O_4_@NC-10 also showed superior NRR performance with a remarkably high NH_3_ yield of 42.58 μg h^−1^ mg_cat_^−1^ and a Faraday efficiency of 8.5% in 0.05 M H_2_SO_4_, which was attributed to the synergistic interaction between the N-doped carbon and the introduced OVs.

### 3.2 Hydrothermal methods

Hydrothermal methods involve chemical reactions in a sealed vessel, where the temperature of the solvent is much higher than its boiling point due to the increase in autogenous pressure caused by heating. The ratio of the raw materials can be adjusted to generate OVs during the hydrothermal process. In recent years this method has been widely used since the powder does not require high-temperature calcination, thus, preventing re-agglomeration of nanoparticles and contamination.

BiVO_4_, containing different concentrations of OVs, was synthesized by Yao et al. by a hydrothermal reaction with adjusted pH values (Figure 2B) (Yao et al., 2019). At pH = 7, BiVO_4_ had the highest concentration of OVs. It showed excellent NRR performance, including NH_3_ yields up to 8.60 μg h^−1^ mg_cat_^−1^, Faraday efficiency of 10.04% at −0.5 V vs RHE. Liu et al. prepared BiVO_4_/TiO_2_ nanotube (BiVO_4_/TNT) heterojunction composites rich in OVs by a hydrothermal method (Liu et al., 2021a). With an NH_3_ yield of 8.54 μg h^−1^ cm^−2^ as well as Faraday efficiency of 7.70% at −0.8 V vs RHE in 0.1 M Na_2_SO_4_, BiVO_4_/TNT exhibited a remarkable performance and showed superior selectivity and high electrochemical stability.

### 3.3 Hydrogen reduction

Hydrogen is a strong reducing agent and is commonly used to reduce metal oxides under high temperature or high pressure to introduce OVs. The concentration of OVs in metal oxides can be controlled effectively by adjusting parameters such as pressure, temperature and gas ratios.

Zhang et al. used MoO_3_ powder as the precursor and employed hydrogen reduction at 900°C for 1 h with different H_2_ proportions (from 5% to 20%) in an Ar-H_2_ atmosphere to obtain MoO_2_ nanosheets with different OVs concentrations (Figure 2C) (Zhang G et al., 2019). DFT calculations showed that appropriately limiting OVs in the MoO_2_ layer benefits the proton transfer step by selectively stabilizing N_2_H* and destabilizing N_2_H_2_* through the distal/alternate mixing path. Consequently, in comparison to MnO_2_ with free OVs, the activation barrier was lowered from 1.49 eV to 0.36 eV. Fang et al. successfully prepared two-dimensional OV-TiO_2_ nanosheets by annealing TiO_2_ nanocrystals in H_2_/Ar at different temperatures (Fang C. H et al., 2019). The OV-TiO_2_-400 nanosheets remained highly stable after 12 cycles and achieved NH_3_ yields of 35.6 μg h^−1^ mg^−1^, which was 2.83 times higher than that of TiO_2_ without OVs.

### 3.4 Wet chemical reduction

Wet chemical reduction of oxides by chemical reagents such as NaBH_4_ can produce OVs at temperatures lower than those required for hydrogen reduction. The reduction molecule first adsorbs on the metal oxide surface and then grabs the O atom to the surface oxygen by electron transfer, thus generating OVs.

Carbon-encapsulated MoO_2_ nanoparticles (MoO_2_@C) with abundant OVs have been synthesized *via* a pectin-assisted hydrothermal method, followed by calcination and treatment with NaBH_4_ solution (Du et al., 2021). MoO_2_@C showed a low NH_3_ yield of 2.12 μg h^−1^ mg^−1^ at −0.7 V vs RHE without treatment with NaBH_4_ solution. However, OV-containing MoO_2_@C showed a higher NH_3_ yield 9.75 μg h^−1^ mg^−1^ at 0.5 V vs RHE and Faraday efficiency of 3.24% and inhibited the HER. Fang et al. synthesized OV-rich TiO_2_ nanoparticles (NPs) grown *in situ* on TiO_2_/Ti_3_C_2_T_*x*_ using an ethanol-based thermal technique (Fang Y. H et al., 2019). Due to the high electrical conductivity of the Ti_3_C_2_T_*x*_ nanosheets, electron transport was promoted and self-aggregation of TiO_2_ nanoparticles was inhibited. Thus, the TiO_2_ nanoparticles increased the surface specificity (SSA) of Ti_3_C_2_T_*x*_. Moreover OVs can serve as NRR reactive sites, the TiO_2_/Ti_3_C_2_T_*x*_ exhibited an excellent NRR capability with NH_3_ yields of 32.17 μg h^−1^ mg^−1^ at −0.55 V vs RHE and Faraday efficiency of 16.07% at −0.45 V vs RHE in 0.1 M HCl. DFT calculations demonstrate that the N≡N triple bond at the TiO_2_ (101)/Ti_3_C_2_T_*x*_ surface was highly activated and showed the lowest NRR energy barrier (0.40 eV) compared to untreated Ti_3_C_2_T_*x*_ or TiO_2_ (101).

### 3.5 Plasma treatment

Efficient and rapid generation of OVs can be achieved by plasma treatment, which involves surface etching and can be carried out at lower temperatures (Wang et al., 2018). Energetic particles, such as various kinds of plasma and high-energy protons, interact with the metal oxide surface during the plasma activation process, the surface structure is damaged, leading to the creation of OVs. The concentration of OVs can be precisely controlled by adjusting the plasma’s power, pressure, gas flow, and irradiation period.

Li et al. used plasma technology to introduce OVs in NiO (Figure 2D) (Li Y. B et al., 2020). DFT calculations show that the electronic structure of NiO was modified due to the introduction of OVs, further improving its electron conduction during NRR, lowering the reaction potential barrier and suppressing side reactions. In contrast to the majority of the reported NRR catalysts, the NiO nanosheets enriched with OVs showed an excellent NH_3_ yield of 29.1 μg h^−1^ mg^−1^ and Faraday efficiency of 10.8% −0.5 V vs. RHE.

### 3.6 Heterogeneous ion doping strategy

Heterogeneous ion doping is based on the difference in the electronegativities of intrinsic atoms, and is used to introduce defects into crystal structures and adjust the physicochemical properties of materials (Li W et al., 2020; Li et al., 2022a). For pure oxygen-containing compounds, both metallic and nonmetallic doping can create an imbalanced charge atmosphere that tends to break the long-term periodicity of the lattice oxygen in the oxide; thus, OVs are formed to maintain thermodynamic stability.

#### 3.6.1 Metal-doping

Chu et al. tuned the NRR properties of CeO_2_ by Fe doping (Fe-CeO_2_) (Chu et al., 2020a). Fe doping transformed the morphology of CeO_2_ from crystalline nanoparticles to partly amorphous nanosheets and significantly increased the concentrations of OVs. As a result of the abundant active sites, considerable specific surface area and high electrical conductivity, Fe-CeO_2_ exhibited good catalytic activity, with an excellent NH_3_ yield of 26.2 μg h^−1^ mg^−1^ (−0.5 V vs. RHE) and Faraday efficiency of up to 14.7% (−0.4 V vs. RHE).

Zhang et al. successfully prepared Cu-doped CeO_2_ nanorods by a facile hydrothermal method, followed by annealing in H_2_/Ar (Zhang S et al., 2019). The synthetic Cu-doped CeO_2_ nanorods were designated as Cu-CeO_2_-*x*, where *x* denotes the amount of Cu dopant. Cu-CeO_2_-3.9 exhibited excellent electrocatalytic performance due to its large surface area of 95.2 m^2^ g^−1^ and mesoporous structure, with NH_3_ yields of 5.3 × 10^–10^ mol s^−1^ cm^−2^ and Faraday efficiencies of 19.1% at −0.45 V vs. RHE in 0.1 M Na_2_SO_4_, which is far beyond that of pure CeO_2_ nanorods. It was found that the Ce^3+^ site in Cu-doped CeO_2_ was more easily replaced by Cu^2+^ with the increase in Cu dopant concentration. As a result, the OVs around the Ce^3+^ sites decreased; conversely, the OVs surrounding the Ce^2+^ sites increased. The OVs formed around the Ce^2+^ sites promoted N_2_ adsorption and activation and improved NRR performance (Figure 2E).

#### 3.6.2 Nonmetal-doping

Chu et al. used a straightforward hydrothermal method to synthesize B-doped MnO_2_ (Chu et al., 2020b). DFT calculations demonstrated that the asymmetric charge distribution brought on by the interaction between OVs and the B dopant enhanced the stability of the crucial intermediate *N_2_H on MnO_2_; thus, lowering the reaction energy barrier and increasing reactivity. the B-MnO_2_/Carbon cloth in 0.5 M LiClO_4_ outperformed most currently known Mn-based catalysts with an NH_3_ yield of 54.2 μg h^−1^ mg^−1^ (−0.4 V vs. RHE) and Faraday efficiency of 16.8% (−0.2 V vs. RHE).

Fan et al. synthesized OV-rich P-doped potassium peroxynitrite (KNb_3_O_8_, abbreviated as P-KNO) by a simple solid-phase method followed by phosphorylation (Fan et al., 2022). The NH_3_ yield of P-KNO was 23.01 μg h^−1^ mg^−1^ (at −0.45 V vs. RHE) and the FE was 39.77% (at −0.4 V vs. RHE) in 0.1 M Na_2_SO_4_ electrolyte (Figure 2F), which is twice that of unphosphorylated KNO. Additionally, due to their complementary effects, P-doping and VOs modified the electronic structure of the catalyst surface, hastening the adsorption and activation of N_2_ and thus enhancing catalytic performance.

## 4 Summary and prospects

Ammonia is one of the most widely manufactured chemicals and has the potential for clean energy applications. However the conventional Haber-Bosch method requires significant amounts of energy and releases a considerable of greenhouse emissions. The efficient, cost-effective, and emission-free ammonia synthesis achieved from N_2_ by ENRR under room temperature has attracted significant research attention. In order to substantially improve the NRR activity, developing and constructing novel effective NRR catalysts is vital.

Oxygen vacancies engineering is an effectively implemented strategy to enhance catalytic activity and selectivity of catalysts by altering the electronic state and creating additional active sites for NRR. In ENRR, OVs can alter the electron density and charge distribution of the catalyst and serve as reaction sites by adsorbing the reactants. By lowering the activation energy barrier, OVs inhibit the HER and increase the efficiency of the NRR.

Although introducing OVs to the catalyst has significantly advanced the development of NRR electrocatalysts, it is still difficult to accurately regulate OVs concentrations and the relationship between OVs and NRR performance is not well understood. In addition, OVs may introduce transition layers in the electrocatalyst during the NRR process, notably in highly acidic and alkaline electrolytes. The reaction pathways are correlated with the properties of the electrolyte and the transition layer, thus, affecting the adsorption of the intermediates and the rate-determining steps. Therefore, it is necessary to consider *in situ* characterization approaches that provide straightforward evidence and in-depth insight into the reaction mechanism. For example, *in situ* FTIR, *in situ* Raman spectroscopy and *in situ* XAFS. The performance of reported catalyst materials is unsuitable for industrial applications and needs to be further improved. We expect that improved, durable and affordable electrocatalysts for NRR can be produced by integrating experiment and theory.
